# Enhancing effects of indirubin on the arsenic disulfide-induced apoptosis of human diffuse large B-cell lymphoma cells

**DOI:** 10.3892/ol.2015.2941

**Published:** 2015-02-09

**Authors:** LING WANG, XIANGLU LI, XINYU LIU, KANG LU, NA CHEN, PEIPEI LI, XIAO LV, XIN WANG

**Affiliations:** 1 Department of Hematology, Provincial Hospital Affiliated to Shandong University, Jinan, Shandong 250021, P.R. China; 2 Department of Hematology, Taian City Central Hospital, Tai’an, Shandong 271000, P.R. China; 3 Institute of Diagnostics, Shandong University, Jinan, Shandong 250012, P.R. China

**Keywords:** diffuse large B-cell lymphoma, indirubin, apoptosis, B-cell lymphoma 2-associated X protein cleavage

## Abstract

The aim of the present study was to investigate the indirubin-enhanced effects of arsenic disulfide (As_2_S_2_) on the proliferation and apoptosis of diffuse large B-cell lymphoma (DLBCL) cells in order to identify an optimum combination therapy. The human DLBCL cells, LY1 and LY8, were treated with different concentrations of indirubin for 24, 48 and 72 h. Next, the cells were treated with 10 μM As_2_S_2_ or a combination of 10 μM As_2_S_2_ and 20 μM indirubin for 48 h. Cell proliferation inhibition was detected using cell counting kit-8 and cell apoptosis was determined using flow cytometry. The expression levels of Bcl-2, Bcl-2-associated X protein (Bax) and caspase-3 were analyzed by quantitative polymerase chain reaction (qPCR) and western blotting. The DLBCL cell viability exhibited no significant changes at 24, 48 or 72 h with increasing indirubin concentration. In addition, the apoptotic rates of the LY1 and LY8 cells demonstrated no noticeable effects at 48 h with increasing indirubin concentration. Following treatment with the combination of indirubin and As_2_S_2_, the inhibitory and apoptotic rates of the cells were notably increased compared with those of the As_2_S_2_-treated group. The qPCR results revealed that indirubin alone had no enhancing effect upon the Bax/Bcl-2 mRNA expression ratio and caspase-3 mRNA expression. Western blot analysis revealed that indirubin alone had an enhancing effect upon the Bax/Bcl-2 protein ratio and procaspase-3 protein expression. In addition, the results demonstrated that the 21-KDa Bax protein was proteolytically cleaved into an 18-KDa Bax in the DLBCL cells treated with the combination of indirubin and As_2_S_2_. Indirubin alone did not inhibit proliferation or induce the apoptosis of the LY1 and LY8 cells. However, the combination of indirubin and As_2_S_2_ yielded enhancing effects. Therefore, the results of the present study demonstrated that with regard to antitumor activities, As_2_S_2_ served as the principal drug, whereas indirubin served as the adjuvant drug. The enhancing effect was due, in part, to the induction of the mitochondrial apoptotic pathway, which involves the cleavage of Bax.

## Introduction

Diffuse large B-cell lymphoma (DLBCL) is the most frequently diagnosed lymphoid tumor, accounting for 40% of all non-Hodgkin lymphomas (NHLs) among adults in Western countries (1). DLBCL is also common in developing countries (2,3). Although traditional chemical therapies and bone marrow transplantations can increase survival rates and even cure certain patients (4), relapse and drug resistance remain a clinical challenge (5).

In order to enhance therapeutic efficacies and reduce adverse effects, multiple drugs are often combined to treat diseases (6). Prescriptions, termed formulae, have been widely used in traditional Chinese medicine (TCM). In general, formulae are composed of several components, including a primary ingredient and additional adjunctive ones. Realgar has demonstrated good anti-tumor effects as part of a Realgar-*Indigo naturalis* formula (RIF); this combines realgar with *indigo naturalis*, *Radix Salvia miltiorrhiza* and *Radix Pseudostellariae heterophylla*. RIF has previously demonstrated effectiveness in the treatment of acute promyelocytic leukemia (APL) (7–10). Further research has indicated that arsenic disulfide (As_2_S_2_) or tetra-arsenic tetra-sulfide (As_4_S_4_) and indirubin, components of realgar and indigo naturalis, are the primary functioning ingredients of RIF (10).

As_2_S_2_ has been used in Western medicine and TCM for several hundred years. Previous studies have demonstrated that realgar is able to induce the apoptosis of a variety of malignant hematological cells (7,10–20). It has also been established that As_4_S_4_ is able to trigger the degradation of the PML-RARα oncoprotein, and indirubin-augmented As_4_S_4_-triggered catabolism of the PML-RARα oncoprotein (10). The findings of our previous study demonstrated that As_2_S_2_ induced the apoptosis of DLBCL cells (21). However, it is yet to be elucidated whether indirubin alone or in combination with As_2_S_2_ exhibits the most significant effects in DLBCL cells. The present study aimed to investigate the indirubin-enhanced effects of As_2_S_2_ on the proliferation and apoptosis of DLBCL cells, and to determine its underlying mechanism in order to identify an optimum combination therapy for the treatment of DLBCL.

## Materials and methods

### Cell lines and cell culture

The LY1 and LY8 cell lines (Albert Einstein College of Medicine, Bronx, NY, USA) were maintained as a suspension at 37°C in 5% carbon dioxide in a humidified atmosphere. The cells were cultured in Iscove’s modified Dulbecco’s medium (IMDM; Hyclone, Logan, UT, USA) supplemented with 10% fetal bovine serum (FBS; Hyclone). Cells in the exponential growth phase were then seeded into 96-well plates or culture flasks.

### Antibodies and reagents

Indirubin (95% in purity) was purchased from Aladdin Reagents Co., Ltd. (Shanghai, China). The indirubin was first dissolved in dimethyl sulfoxide to generate a 20-M stock solution. Next, the stock solution was passed through a 0.22-μm filter. The aliquots were further diluted in IMDM supplemented with 10% FBS prior to each experiment in order to prepare the working solutions. The monoclonal rabbit anti-human B-cell lymphoma 2 (Bcl-2; 1:1,000), rabbit anti-human Bcl-2-associated X protein (Bax; 1:1,000) and rabbit anti-human caspase-3 (1:1,000) antibodies were purchased from Cell Signaling Technology, Inc. (Danvers, MA, USA). The polyclonal mouse anti-human β-actin (1:1,000) antibody was obtained from the Zhongshan Goldenbridge Biotechnology Co., Ltd. (Beijing, China).

### Assessment of cellular cytotoxicity using the cell counting kit-8 (CCK-8)

Cell proliferation was analyzed using the CCK-8 assay (Beyotime Institute of Biotechnology, Haimen, China). In total, 1×10^4^ cells/100 μl/well of the LY1 and LY8 cells were seeded into 96-well plates, treated with various concentrations of indirubin (1, 5, 10, 15 and 20 μM), and then cultured for 24, 48 and 72 h in a humidified atmosphere in a 5% carbon dioxide incubator. Next, the cells were treated with 10 μM As_2_S_2_ (99.53% purity, Alfa Aesar Company, Shanghai, China) and 20 μM indirubin, alone or in combination, for 48 h. Each experiment was performed in triplicate. The cells were then incubated with 10 μl CCK-8 at 37°C for 4 h. An ELISA reader (Spectra Max M2; Molecular Devices LLC, Sunnyvale, CA, USA) was then used to measure the optical density of each well at 450 nm.

### Assessment of apoptosis using Annexin V and propidium iodide (PI)

The apoptotic rate of the LY1 and LY8 cells was analyzed using an Annexin V-fluorescein isothiocyanate (FITC) apoptosis detection kit (KeyGen Biotech Co., Ltd., Nanjing, China). First, the cells were treated with different concentrations of indirubin and As_2_S_2_, alone or in combination, for 48 h. Dual staining with Annexin V-FITC and PI was then performed according to the manufacturer’s instructions. In total, 5–10×10^5^ cells were analyzed using a flow cytometer (BD Biosciences, Franklin Lakes, NJ, USA). FlowJo 7.6 software (FlowJo LLC, Ashland, OR, USA) was then used to process the data. The cells that were negative for the Annexin V-FITC and PI stain were identified as viable cells. The cells that were positive for Annexin V-FITC, but negative for PI were considered to be early apoptotic cells, while those with positive Annexin V-FITC and PI staining were identified to be late apoptotic cells. The sum of the early and late apoptotic cells constituted the total number of apoptotic cells.

### Gene expression study using quantitative polymerase chain reaction (qPCR)

The total RNA was extracted using TRIzol (Invitrogen, Carlsbad, CA, USA) from the DLBCL cells that had been treated with indirubin and As_2_S_2_, alone or in combination, and from the untreated cells. The reverse transcription reaction step was then performed using Takara reverse transcription reagents (Takara, Dalian, China). The amplification reactions were performed using SYBR Premix Ex Taq (Takara) on a Roche LightCycler 480 Real-Time PCR System (Roche Diagnostics, Basel, Switzerland). Specific primers for the qPCR were purchased from Sangon Biotech Co., Ltd. (Shanghai, China). The primer sequences are listed in Table I. In order to control the variability in expression levels, the data were normalized to the geometric mean of the housekeeping gene, β-actin. For the data analysis, the 2^−ΔΔCt^ method was used. The qPCR for each gene of each cDNA sample was performed in triplicate.

$$\begin{array}{c} \Delta \text{Ct}=\text{Ct }(\text{target gene})-\text{Ct }(\beta \text{-actin gene})\\ \Delta \Delta \text{Ct}=\Delta \text{Ct }(\text{drug-treated cells})-\Delta \text{Ct }(\text{untreated control}) \end{array}$$

### Protein expression analysis using western blotting

SDS-PAGE and western blot analysis were performed in order to evaluate the protein levels of Bax, Bcl-2 and caspase-3. The total protein was extracted from the DLBCL cells treated with indirubin and As_2_S_2_, alone or in combination, and from the untreated cells, using a radioimmunoprecipitation assay and 1% phenylmethylsulfonyl fluoride (Shenergy Biocolor Bioscience and Technology Co., Ltd., Shanghai, China). Next, the bicinchoninic acid assay (Shenergy Biocolor Bioscience and Technology Co., Ltd.) was used to determine the protein concentration of the samples. The proteins were detected using a chemiluminescence detection kit (EMD Millipore, Billerica, MA, USA). The results of the western blot analysis were analyzed using ImageQuant Las 4000 software (GE Healthcare Life Sciences, Shanghai, China) and Multi Gauge version 3.0 software (Fujifilm Life Science, Tokyo, Japan).

### Statistical analysis

Statistical analysis was performed using SPSS version 17.0 software (SPSS, Inc., Chicago, IL, USA). The data are presented as the mean ± standard deviation. An analysis of variance was used to evaluate the data from the cellular viability and apoptosis assays. Additional statistical analyses were performed using Student’s t-test. P<0.05 was used to indicate a statistically significant difference.

## Results

### Effects of indirubin and As_2_S_2_, alone or in combination, on the viability of DLBCL cells

**Figure 1:**
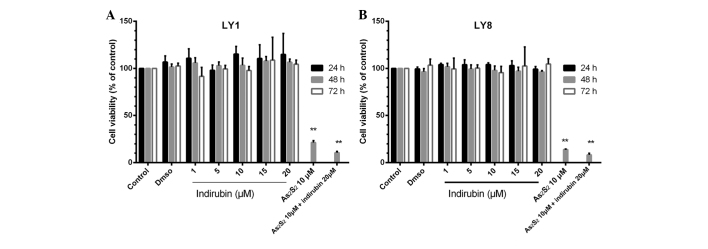
Effects of indirubin and arsenic disulfide (As_2_S_2_), alone or in combination, on the viability of diffuse large B-cell lymphoma cells. (A) LY1 and (B) LY8 cells were incubated with different doses of indirubin (1, 5, 10, 15 and 20 μM) for 24, 48 and 72 h. In addition, (A) LY1 and (B) LY8 cells were incubated and 10 μM As_2_S_2_ and 20 μM indirubin, alone or in combination, for 48 h. The cell counting kit-8 assay was used to evaluate the cell viability. The presented data includes the corresponding untreated control. Values are expressed as the mean ± standard deviation. ^**^P<0.01 vs. untreated control. DMSO, dimethyl sulfoxide.

The effect of indirubin and As_2_S_2_, alone or in combination, on the viability of the LY1 and LY8 cells was investigated. The results demonstrated that there was no significant effect on the viability of the cells following incubation with different doses of indirubin (1, 5, 10, 15 and 20 μM) for different lengths of time (24, 48 and 72 h) (Fig. 1). Compared with the untreated control group, treatment with 10 μM As_2_S_2_ for 48 h resulted in a cell viability of 21.30±2.10% and 13.89±0.46% for the LY1 and LY8 cells, respectively (P<0.01). The inhibitory effect on cell viability was enhanced following treatment with the combination of 10 μM As_2_S_2_ and 20 μM indirubin. The cell viability rate in the combination group was 10.56±1.27 and 8.20±1.70% for the LY1 and LY8 cells, respectively (P<0.01). Significant differences were identified between the cell viability rate of the As_2_S_2_-treated group and the combination-treated group (P<0.01).

### Effects of indirubin and As_2_S_2_, alone or in combination, on the apoptosis of DLBCL cells

**Figure 2:**
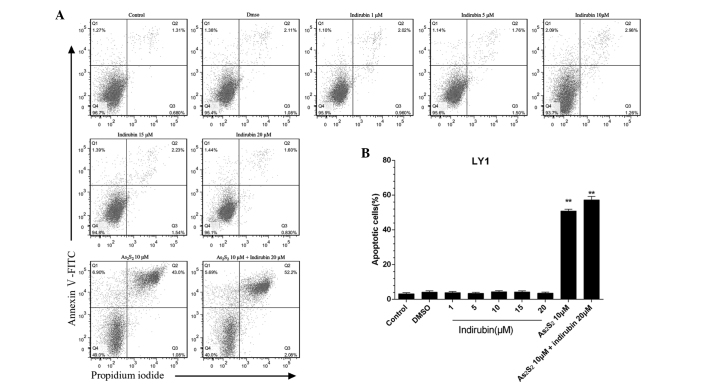
Effects of indirubin and arsenic disulfide (As_2_S_2_), alone or in combination, on the apoptosis of LY1 cells. LY1 cells were incubated with different doses of indirubin (1, 5, 10, 15 and 20 μM) and also 10 μM As_2_S_2_ and 20 μM indirubin, alone or in combination, for 48 h. (A) The apoptotic rate was determined using Annexin V-fluorescein isothiocyanate (FITC)/propidium iodide (PI) dual staining followed by flow cytometric analysis. The lower left quadrant (Q4) indicates the percentage of viable cells (Annexin V-FITC- and PI-negative), the upper left quadrant (Q1) indicates the percentage of early apoptotic cells (Annexin V-FITC-positive and PI-negative) and the upper right quadrant (Q2) indicates the percentage of late apoptotic cells (Annexin V-FITC- and PI-positive). (B) There were no significant effects on the rate of apoptosis of the LY1 cells following incubation with different doses of indirubin. However, significant differences were evident between the As_2_S_2_-treated group and the combination-treated group. Values are expressed as the mean ± standard deviation. ^**^P<0.01 vs. untreated control. DMSO, dimethyl sulfoxide.

**Figure 3:**
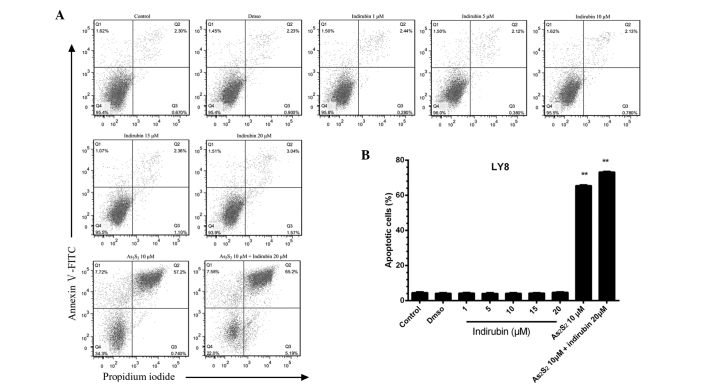
Effects of indirubin and arsenic disulfide (As_2_S_2_), alone or in combination, on the apoptosis of LY8 cells. LY8 cells were incubated with different doses of indirubin (1, 5, 10, 15 and 20 μM) and also 10 μM As_2_S_2_ and 20 μM indirubin, alone or in combination, for 48 h. (A) The apoptotic rate was determined using Annexin V-fluorescein isothiocyanate (FITC)/propidium iodide (PI) dual staining followed by flow cytometric analysis. The lower left quadrant (Q4) indicates the percentage of viable cells (Annexin V-FITC- and PI-negative), the upper left quadrant (Q1) indicates the percentage of early apoptotic cells (Annexin V-FITC-positive and PI-negative) and the upper right quadrant (Q2) indicates the percentage of late apoptotic cells (Annexin V-FITC- and PI-positive). (B) There were no significant effects on the rate of cell apoptosis of the LY8 cells following incubation with different doses of indirubin. However, significant differences were evident between the As_2_S_2_-treated group and the combination-treated group. Values are expressed as the mean ± standard deviation. ^**^P<0.01 vs. untreated control. DMSO, dimethyl sulfoxide.

The effects of the indirubin-induced apoptosis on the LY1 and LY8 cells were assessed. As shown in Figs. 2 and 3, no significant effects were identified on the apoptosis of the LY1 and LY8 cells following treatment with different doses of indirubin (1, 5, 10, 15 and 20 μM) for 48 h. Compared with the untreated control group, treatment with 10 μM As_2_S_2_ for 48 h resulted in an apoptotic cell rate of 50.86±1.01 and 65.42±0.47% for the LY1 and LY8 cells, respectively (P<0.01). The apoptotic cell rate also increased following treatment with the combination of 10 μM As_2_S_2_ and 20 μM indirubin. The cell apoptosis rate of the combination group was 57.26±1.99 and 73.19±0.40% for the LY1 and LY8 cells, respectively (P<0.01). Significant differences were identified between the As_2_S_2_-treated group and the combination-treated group (P<0.01).

### Effects of indirubin and As_2_S_2_, alone or in combination, on the transcription levels of Bax, Bcl-2 and caspase-3 genes in DLBCL cells

**Figure 4:**
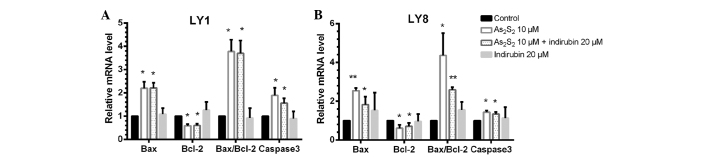
Effects of indirubin and arsenic disulfide (As_2_S_2_), alone or in combination, on the transcription levels of B-cell lymphoma 2 (Bcl-2), Bcl-2-associated X protein (Bax) and caspase-3 genes in diffuse large B-cell lymphoma cells. The relative mRNA levels of Bax, Bcl-2 and caspase-3 genes were assessed by quantitative polymerase chain reaction following treatment with 10 μM As_2_S_2_ and 20 μM indirubin, alone or in combination, for 48 h in (A) LY1 and (B) LY8 cells. Values are expressed as the mean ± standard deviation. ^*^P<0.05 and ^**^P<0.01 vs. untreated control.

Our previous study demonstrated that As_2_S_2_-induced apoptosis was dependent upon the mitochondrial-mediated apoptosis pathway (21). In order to investigate whether the combination treatment led to enhancement of the mRNA levels of the Bax, Bcl-2 and caspase-3 genes, qPCR was performed. As shown in Fig. 4, no significant differences in the expression of the Bax, Bcl-2 and caspase-3 genes in the DLBCL cells were observed between the As_2_S_2_ group and the combination group.

### Effects of indirubin and As_2_S_2_^,^ alone or in combination, on the protein levels of Bax, Bcl-2 and caspase-3 genes in DLBCL cells

**Figure 5:**
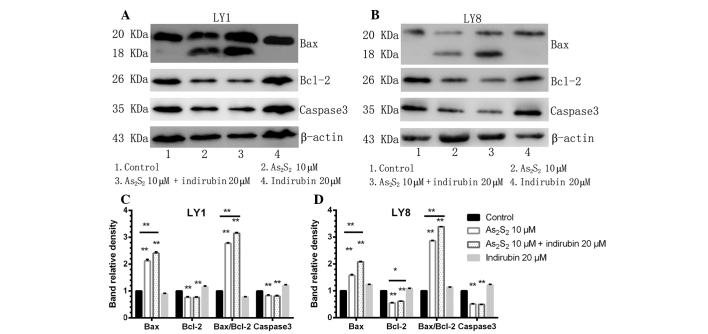
Effects of indirubin and arsenic disulfide (As_2_S_2_), alone or in combination, on the levels of B-cell lymphoma 2 (Bcl-2), Bcl-2-associated X protein (Bax) and caspase-3 protein in diffuse large B-cell lymphoma cells. (A) LY1 and (B) LY8 cells were treated with 10 μM As_2_S_2_ and 20 μM indirubin, alone or in combination, for 48 h. Western blotting was used to analyze whole cell lysates for Bax, Bcl-2 and caspase-3. β-actin expression was used as an internal control. The relative density of caspase-3 and the Bax/Bcl-2 ratio in (C) LY1 and (D) LY8 cells was calculated from three separate experiments. ^*^P<0.05 and ^**^P<0.01 vs. untreated control group.

The protein expression levels of Bax, Bcl-2 and caspase-3 were investigated. The protein levels following treatment are shown in Fig. 5. Subsequent to treatment for 48 h, the expression of Bcl-2 was markedly downregulated, whereas the expression of Bax was notably upregulated in the LY1 and LY8 cells of the combination group compared with the control. Furthermore, the Bax/Bcl-2 ratio was significantly increased. Our previous study demonstrated that following exposure to 10 μM As_2_S_2_, the levels of the 21-KDa Bax protein and the 18-KDa Bax cleavage protein were markedly increased (21). Western blot analysis also revealed that following treatment, the levels of the 21-KDa Bax protein and the cleaved 18-KDa Bax protein were elevated in the combination group. By contrast, procaspase-3 expression was evidently reduced.

## Discussion

Previous studies have demonstrated that indirubin and its derivatives inhibit the proliferation and induce the apoptosis of a number of malignant cells (22–50). Previous studies have also demonstrated that indirubin derivatives induce G_2_/M arrest by inhibiting cyclin-dependent kinases and inducing apoptosis via the mitochondria-dependent pathway (49,51,52). In human malignant B (IM9 and Reh6) and T (Jurkat and CEM-T) cells, indirubin-3-oxime and 6-bromo-indirubin-3-monoxime were shown to initiate cell cycle inhibition and apoptosis. In the same way, meisoindigo inhibited the proliferation and induced the apoptosis of Jurkat cells in a dose-dependent manner. In addition to the onset of apoptosis, caspases 2, 3, 8 and 9 were activated and AKT phosphorylation was decreased in Jurkat cells following meisoindigo treatment. A study by Kim *et al* revealed that the indirubin derivative, 5-fluoro-indirubin, had a synergic effect on 1,25-dihydroxyvitamin D3 (1,25-(OH)_2_D_3_)- and all-*trans* retinoic acid-induced differentiation HL-60 leukemia cells (53).

In a previous study, indirubin derivatives demonstrated biphasic effects in prostate cells. The derivatives stimulated the growth of androgen-dependent prostate cancer cells at sub-apoptotic concentrations, but also inhibited the proliferation and induced the apoptosis of prostate cancer cells at higher concentrations, causing cell toxicity and apoptosis (54). The results of the present study revealed that indirubin alone had no effect upon the proliferation and apoptosis of the DLBCL cells. However, when combined with As_2_S_2_, indirubin had an enhancing effect upon the proliferation and apoptosis of the cells, which was consistent with the findings of previous studies. Wang *et al* revealed that indirubin alone had no effect on the differentiation of APL cells, but that it could enhance As_4_S_4_-induced differentiation. Furthermore, although indirubin did not cause degradation of the PML-retinoic acid receptor α (PML/RARα) oncoprotein, it enhanced the As_4_S_4_-triggered degradation of PML/RARα and initiated ubiquitination (10). Therefore, indirubin served as an adjuvant ingredient for the treatment of APL.

It is well known that apoptosis is key to the development and maintenance of homeostasis in multiple organisms. The results of our previous study suggested that As_2_S_2_ inhibited the proliferation and induced the apoptosis of DLBCL cells *in vitro* via the mitochondrial pathway (21). Bax was revealed to be important for the initiation of apoptosis in the As_2_S_2_-treated DLBCL cells. The results of the present study demonstrated that the combination of As_2_S_2_ and indirubin notably enhanced the apoptosis of the DLBCL cells. Such findings should encourage further studies to investigate the value of this TCM formula. Future studies that investigate indirubin derivatives with higher water-solubility are required in order to identify a novel formula with improved antitumor characteristics.

In conclusion, indirubin alone did not inhibit the proliferation or induce the apoptosis of the DLBCL cells. However, the combination of indirubin and As_2_S_2_ yielded enhancing effects. Therefore, the results of the present study suggested that As_2_S_2_ served as the principal drug and that indirubin served as the adjuvant drug. The enhancing effect was due, in part, to the induction of the mitochondria-dependent apoptotic pathway, which involves the cleavage of Bax.

## Figures and Tables

**Table I:** Primers used for the quantitative polymerase chain reaction.

Gene	Primer sequence	Product, bp
β-actin	Forward: 5′-TGACGTGGACATCCGCAAAG-3′ Reverse: 5′-CTGGAAGGTGGACAGCGAGG-3′	205
Bax	Forward: 5′-CCCGAGAGGTCTTTTTCCGAG-3′ Reverse: 5′-CCAGCCCATGATGGTTCTGAT-3′	155
Bcl-2	Forward: 5′-ATGTGTGTGGAGAGCGTCAA-3′ Reverse: 5′-ACAGTTCCACAAAGGCATCC-3′	136
Caspase-3	Forward: 5′-GACTCTGGAATATCCCTGGgACAACA-3′ Reverse: 5′-AGGTTTGCTGCATCGACATCTG -3′	140

Bcl-2, B-cell lymphoma 2; Bax, Bcl-2-associated X protein.
